# Association of Major Food Sources of Fructose-Containing Sugars With Incident Metabolic Syndrome: A Systematic Review and Meta-analysis

**DOI:** 10.1001/jamanetworkopen.2020.9993

**Published:** 2020-07-09

**Authors:** Zhila Semnani-Azad, Tauseef A. Khan, Sonia Blanco Mejia, Russell J. de Souza, Lawrence A. Leiter, Cyril W. C. Kendall, Anthony J. Hanley, John L. Sievenpiper

**Affiliations:** 1 Department of Nutritional Sciences, Faculty of Medicine, University of Toronto, Toronto, Ontario, Canada; 2 Toronto 3D Knowledge Synthesis and Clinical Trials Unit, Risk Factor Modification Centre, St Michael’s Hospital, Toronto, Ontario, Canada; 3 Department of Health Research Methods, Evidence, and Impact, Faculty of Health Sciences, McMaster University, Hamilton, Ontario, Canada; 4 Population Health Research Institute, Hamilton, Ontario, Canada; 5 Division of Endocrinology and Metabolism, University of Toronto, Toronto, Ontario, Canada; 6 Li Ka Shing Knowledge Institute, St Michael’s Hospital, Toronto, Ontario, Canada; 7 Division of Nutrition and Dietetics, College of Pharmacy and Nutrition, University of Saskatchewan, Saskatoon, Saskatchewan, Canada; 8 Leadership Sinai Centre for Diabetes, Mount Sinai Hospital, Toronto, Ontario, Canada

## Abstract

**Importance:**

Sugar-sweetened beverages (SSBs) are associated with increased risk of metabolic syndrome (MetS). However, the role of other important food sources of fructose-containing sugars in the development of MetS remains unclear.

**Objective:**

To examine the association of major food sources of fructose-containing sugars with incident MetS.

**Data Sources:**

MEDLINE, Embase, and Cochrane Library were searched from database inception to March 24, 2020, in addition to manual searches of reference lists from included studies using the following search terms: *sugar-sweetened beverages*, *fruit drink*, *yogurt*, *metabolic syndrome*, and *prospective study*.

**Study Selection:**

Inclusion criteria included prospective cohort studies of 1 year or longer that investigated the association of important food sources of fructose-containing sugars with incident MetS in participants free of MetS at the start of the study.

**Data Extraction and Synthesis:**

Study quality was assessed using the Newcastle-Ottawa Scale. Extreme quantile risk estimates for each food source with MetS incidence were pooled using a random-effects meta-analysis. Interstudy heterogeneity was assessed (Cochran *Q* statistic) and quantified (*I*^2^ statistic). Dose-response analyses were performed using a 1-stage linear mixed-effects model. The certainty of the evidence was assessed using GRADE (Grading of Recommendations, Assessment, Development, and Evaluation). Results were reported according to the Meta-analysis of Observational Studies in Epidemiology (MOOSE) and Preferred Reporting Items for Systematic Reviews and Meta-analyses (PRISMA) reporting guidelines.

**Main Outcomes and Measures:**

Pooled risk ratio (RR) of incident MetS (pairwise and dose response).

**Results:**

Thirteen prospective cohort studies (49 591 participants [median age, 51 years; range, 6-90 years]; 14 205 with MetS) that assessed 8 fructose-containing foods and MetS were included. An adverse linear dose-response association for SSBs (RR for 355 mL/d, 1.14; 95% CI, 1.05-1.23) and an L-shaped protective dose-response association for yogurt (RR for 85 g/d, 0.66; 95% CI, 0.58-0.76) and fruit (RR for 80 g/d, 0.82; 95% CI, 0.78-0.86) was found. Fruit juices (mixed and 100%) had a U-shaped dose-response association with protection at moderate doses (mixed fruit juice: RR for 125 mL/d, 0.58; 95% CI, 0.42-0.79; 100% fruit juice: RR for 125 mL/d, 0.77; 95% CI, 0.61-0.97). Honey, ice cream, and confectionary had no association with MetS incidence. The certainty of the evidence was moderate for SSBs, yogurt, fruit, mixed fruit juice, and 100% fruit juice and very low for all other food sources.

**Conclusions and Relevance:**

The findings of this meta-analysis suggest that the adverse association of SSBs with MetS does not extend to other food sources of fructose-containing sugars, with a protective association for yogurt and fruit throughout the dose range and for 100% fruit juice and mixed fruit juices at moderate doses. Therefore, current policies and guidelines on the need to limit sources of free sugars may need to be reexamined.

## Key Points

**Question:**

What is the association of major food sources of fructose-containing sugars with incident metabolic syndrome?

**Findings:**

In this systematic review and meta-analysis of 13 prospective studies including 49 591 participants, the adverse association of sugar-sweetened beverages with incident metabolic syndrome did not extend to other major food sources of fructose-containing sugars. Yogurt, fruit, 100% fruit juice, and mixed fruit juice all had a protective association with incident metabolic syndrome.

**Meaning:**

Generalized statements on the adverse effects of fructose-containing sugars cannot be extrapolated from sugar-sweetened beverage results, as assessment of other important food sources of fructose-containing sugars show protective associations with metabolic syndrome incidence.

## Teaser

This systematic review and meta-analysis examines the association of major food sources of fructose-containing sugars with incident metabolic syndrome.

## Introduction

Metabolic syndrome (MetS) is a cluster of major health risk factors associated with an increased incidence of type 2 diabetes and cardiovascular disease.^1^ Although the definition and criteria for identifying MetS can vary,^2,3^ all definitions consider important risk factors, including large waist circumference, elevated blood pressure, low high-density lipoprotein level, elevated levels of triglycerides, and hyperglycemia.

Fructose-containing sugars (eg, sucrose and high-fructose corn syrup) in the diet have been implicated as potential contributing factors to increased MetS risk.^4,5^ There is strong evidence that sugar-sweetened beverages (SSBs), a major source of fructose in the North American diet, are associated with increased incident MetS.^6^ The role of other important food sources of fructose-containing sugars in the development of MetS, however, has yet to be fully elucidated. This systematic review and dose-response meta-analysis of prospective cohort studies examines the association of food sources of fructose-containing sugars and incident MetS and evaluates the strength and quality of the evidence using GRADE (Grading of Recommendations, Assessment, Development, and Evaluation).^7^

## Methods

### Data Sources and Searches

This meta-analysis followed the *Cochrane Handbook for Systematic Reviews of Interventions*.^8^ Results were reported according to the Meta-analysis of Observational Studies in Epidemiology (MOOSE) and Preferred Reporting Items for Systematic Reviews and Meta-analyses (PRISMA) reporting guidelines.^9,10^ The study protocol was registered at ClinicalTrials.gov.^11^ Data sources included MEDLINE, Embase, and the Cochrane Library from database inception to March 24, 2020. Manual search of the reference lists from included studies supplemented the database search. Search terms reflected the most consumed food sources of fructose-containing sugars (based on national surveys that outlined the leading fructose-containing added or free sugar foods^12,13,14^) (eg, *sugar-sweetened beverages*, *fruit drink*, and *yogurt*), the outcome of interest (eg, *metabolic syndrome*), and the study design (eg, *prospective study*) (eTable 1 in the Supplement).

### Study Selection

Prospective cohort studies of 1 year or longer that investigated the association of major food sources of fructose-containing sugars with incident MetS in participants free of MetS at baseline were included (eTable 1 in the Supplement). If multiple publications of the same cohort provided results on the same outcome with overlapping groups of individuals, the longest follow-up study was included. Abstracts and unpublished studies were excluded.

### Data Extraction and Quality Assessment

Two independent reviewers (Z.S.A. and T.A.K) extracted relevant data, including sample size, participant characteristics, food source of fructose-containing sugars, exposure levels, follow-up duration, number of MetS cases, covariates in fully adjusted models, and the relative risk (RR) with 95% CIs of incident MetS per category of intake, median dose in each category, and funding source, dual-sequentially. Studies were assessed for risk of bias using the Newcastle-Ottawa Scale.^15^ Newcastle-Ottawa Scale points were awarded based on cohort selection, adequacy of outcome measures, and comparability of cohorts regarding design or analysis.^15^ A maximum of 9 points could be awarded, with 0 points indicating lowest study quality and 9 points indicating highest study quality. A score of 6 points was the minimum threshold for the study to be considered higher quality.^16^ Disagreements were resolved by consensus or by involving a third person (J.L.S.). The GRADE approach was used to assess the overall certainty and strength of the evidence, ranging from high to very low certainty (eAppendix 1 in the Supplement).^7^

### Statistical Analysis

Pairwise meta-analyses and sensitivity analyses were conducted in R software, version 3.6.1 (R Foundation for Statistical Computing) using dmetar.^17^ Dose-response analyses were conducted in Stata software, version 16 (StataCorp) using drmeta.^18^ Each food source of fructose-containing sugar was considered as an independent exposure. Risk ratios (RRs) of extreme quantiles from the most adjusted models were used for pairwise analyses.^8^ When studies used continuous RRs per dose, we imputed the extreme quantiles from other publications of the same or similar cohort. Hazard ratios and odds ratios were converted to RRs based on the recommended method by Zhang and Yu^19^ (eAppendix 2 in the Supplement).

Summary estimates were determined by natural log transforming and pooling the RRs using the DerSimonian and Laird random-effects model.^20^ A fixed-effects model was used if the number of studies was 5 or fewer.^21^ Unit-of-analysis error (for studies that appeared more than once in the same food source analysis) was addressed by dividing participants equally among the multiple comparisons and readjusting the log SEs.^8^ Interstudy heterogeneity was assessed using the Cochran *Q* (χ^2^) statistic and quantified by the *I*^2^ statistic, where *I*^2^ of 50% or greater and *P* < .10 determined by the *Q* statistic represented evidence of substantial heterogeneity.^8^ Sources of heterogeneity were assessed by sensitivity analyses that involved the systematic removal of each study for food sources with more than 2 cohorts. If 10 or more cohort comparisons were available, a priori subgroup analyses were performed.

If 10 or more cohort comparisons were available, studies were assessed for publication bias by visual inspection of funnel plots and formal testing using the Begg and Egger tests,^22,23^ with significance set at *P* < .10. In the presence of publication bias, the Duval and Tweedie trim and fill method was used.^24^

Dose responses were modeled using RRs (95% CIs) from dose categories to determine the shape of the association between the dose of the fructose-containing foods and the risk of MetS (eAppendix 3 in the Supplement).^25,26^ Doses were defined as the mean consumption in each reported category or quantile. We reported nonlinear associations for a study if results of the Wald test for departure from linearity were significant at *P* < .10 (2-sided).^27^ The significance for the main pooled RR for the pairwise analyses was based on *P* < .05.

## Results

### Search Results

**Figure 1.**
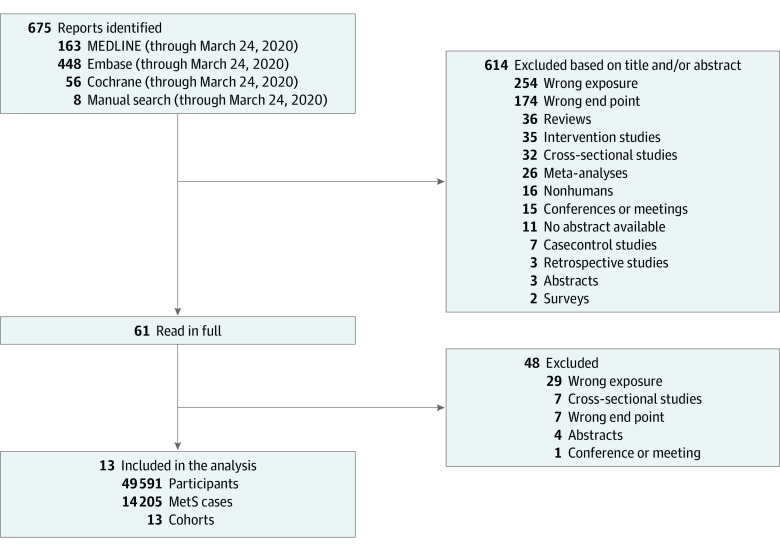
Diagram of Study Selection MetS indicates metabolic syndrome.

Thirteen reports^28,29,30,31,32,33,34,35,36,37,38,39,40^ (49 591 participants and 14 205 cases) with data from 8 unique prospective cohorts met the inclusion criteria (Figure 1). Eight major food sources of fructose-containing sugars were identified, including SSBs (7 cohort comparisons; 20 480 participants and 7406 cases^28,32,34,36,37,38^), mixed fruit juice (3 cohort comparisons; 3062 participants and 1322 cases^32,33,38^), 100% fruit juice (2 cohort comparisons; 5464 participants and 1389 cases^31,32,40^), fruit (4 cohort comparisons; 10 074 participants and 3002 cases^30,33,40^), yogurt (5 cohort comparisons; 19 057 participants and 3877 cases^29,30,35,39^), honey (1 cohort; 3616 participants and 590 cases^30^), ice cream (1 cohort; 3616 participants and 590 cases^30^), and confectionary (2 cohort comparisons; 1476 participants and 250 cases^30^). Prospective cohort studies that assessed grain and grain-based products or other fruit- or dairy-based products with incident MetS were not identified.

### Study Characteristics

The Table gives the characteristics of the 13 prospective cohort studies.^28,29,30,31,32,33,34,35,36,37,38,39,40^ Studies included data from the US,^28,31,36^ Spain,^29,32,39^ Iran,^30,37,38^ and South Korea.^33,34,35,40^ Participants ranged from adolescents to older adults (median age, 51 years; range, 6-90 years). Appelhans et al^28^ exclusively studied a female cohort. The mean (SD) duration of follow-up was 5.7 (3.3) years (range, 2.0-14.0 years). Fruit juice was considered to be mixed fruit juice if the study combined fruit drinks and fruit juice or did not specify the kind of fruit juice (100% fruit juice or fruit drink). Yogurt was considered a source of fructose given that more than 70% of the yogurt products are flavored^41^ and consumers prefer yogurt products with a moderate (approximately 7%-10%) concentration of added sucrose.^42,43,44^ MetS was defined using the Adult Treatment Panel III,^31,34,35,36,37,38,40^ harmonized criteria,^28,29,30,32,39^ or a continuous scale^33^ (eAppendix 4 in the Supplement). All studies were agency funded.

**Table.** Characteristics of Prospective Cohort Studies Investigating Dietary Intake of Food Sources of Fructose-Containing Sugars and MetS

Source	Cohort name	Country	Follow-up duration, y	Sex	No. of participants	No. of MetS cases	Baseline age range, y	Dietary assessment	Food source	MetS assessment	Funding source
Appelhans et al,^28^ 2017	SWAN	US	14^a^	Female	1401	268	42-52	FFQ (interviewer administered)	SSB	Harmonized criteria^c^	Agency ^d^
Babio et al,^29^ 2015	PREDIMED	Spain	3.2^b^	Both	1868	930	Male: 55-80; female: 60-80,	SFFQ	Yogurt	Harmonized criteria	Agency
Cheraghi et al,^30^ 2016	TLGS	Iran	2.05^b^	Both	3616	590	≥20	FFQ (interviewer administered)	Fruit, yogurt, ice cream, honey	Harmonized criteria	Agency
Duffey et al,^31^ 2010	CARDIA	US	7^a^	Both	3596	459	18-30	SFFQ (interviewer administered)	100% fruit juice	ATP III	Agency
Ferreira-Pêgo et al,^32^ 2016	PREDIMED	Spain	3.24^b^	Both	1868	930	55-80	SFFQ	SSB, mixed fruit juice,100% fruit juice	Harmonized criteria	Agency
Hur et al,^33^ 2016	KoCAS	South Korea	4^a^	Both	770	345	9-10	3-d FR	Fruit sugar, beverage sugar	cMET	Agency
Kang and Kim,^34^ 2017	KoGES	South Korea	5.7^a^	Both	5797	2129	40-69	SFFQ	SSB	ATP III	Agency
Kim and Kim,^35^ 2017	KoGES	South Korea	5.7^a^	Both	5510	2103	40-69	SFFQ	Yogurt	ATP III	Agency
Lim and Kim,^40^ 2019	KoGES	South Korea	8^a^	Both	5688	2067	40-69	SFFQ	Fruit	ATP III	Agency
Lutsey et al,^36^ 2008	ARIC	US	9^a^	Both	9514	3782	45-64	FFQ	SSB	ATP III	Agency
Mirmiran et al,^37^ 2014	TLGS	Iran	3^a^	Both	1476	249	19-70	SFFQ	Biscuits and cakes, candies and chocolate, SSB	ATP III with specific waist circumference cutoffs for Iranian adults	Agency
Mirmiran et al,^38^ 2015	TLGS	Iran	3.6^a^	Both	424	47	6-18	SFFQ	SSB, mixed fruit juice	ATP III adapted definition for adolescents	Agency
Sayón-Orea et al,^39^ 2015	SUN	Spain	6^a^	Both	8063	306	20-90	SFFQ	Yogurt	Harmonized criteria	Agency

Abbreviations: ARIC, Atherosclerosis Risk in Communities Study; ATP III, Adult Treatment Panel III; CARDIA, Coronary Artery Risk Development in Young Adults; cMET, continuous MetS score; FFQ, Food Frequency Questionnaire; FR, food records; KoCAS, Korean Child-Adolescent Cohort Study; KoGES, Korean Genome and Epidemiology Study; MetS, metabolic syndrome; PREDIMED, Prevención con Dieta Mediterránea; TLGS, Tehran Lipid and Glucose Study; SFFQ: Semiquantitative Food Frequency Questionnaire; SSB, sugar-sweetened beverage; SUN, Sequimiento University of Navarra; SWAN, Study of Women’s Health Across the Nation.

^a^ Mean value.

^b^ Median value.

^c^ Harmonized criteria of the American Heart Association/National Heart, Lung, and Blood Institute, and the International Diabetes Federation definitions for metabolic syndrome.

^d^ Agency funding is that from government, university, or not-for-profit health agency.

All studies,^28,29,31,32,33,34,35,36,37,38,39,40^ except for the study by Cheraghi et al,^30^ adjusted for age and multiple prespecified primary confounding variables, including sex, markers of obesity, smoking, family history of MetS, energy or calorie intake, diabetes, physical activity, and alcohol intake (eTable 2 in the Supplement). Between 4 and 26 variables were adjusted for in fully adjusted models of the 12 studies^28,29,31,32,33,34,35,36,37,38,39,40^ that detailed their statistical process.

### Risk of Bias

None of the studies were rated as high risk of bias (eTable 3 in the Supplement). Statistical tests for publication bias could not be assessed for any food source because of 10 or fewer cohort comparisons.

### Important Food Sources of Fructose-Containing Sugars and Incident MetS

**Figure 2.**
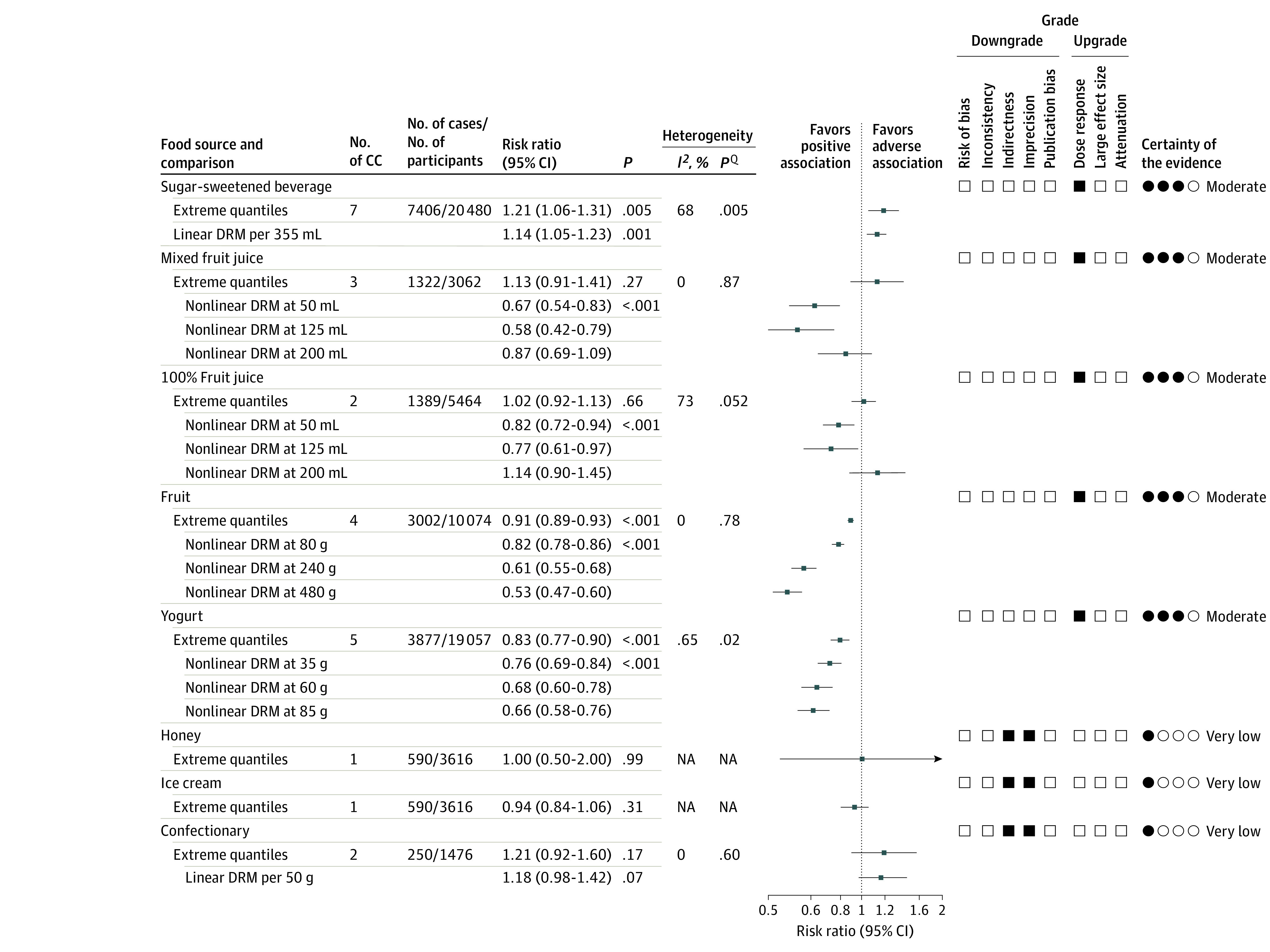
Summary Superplot for the Association Between Important Food Sources of Fructose-Containing Sugars and Incident Metabolic Syndrome Pooled risk estimate is represented by the data markers. *I*^2^ values of 50% or greater (*P* < .10 determined by the *Q* statistic) indicate substantial heterogeneity,^45^ and risk ratios greater than 1.00 indicate an adverse association. The Grading of Recommendations, Assessment, Development, and Evaluation (GRADE) of prospective cohort studies are rated as low certainty of evidence and can be downgraded by 5 domains and upgraded by 3 domains. The filled black squares indicate downgrade and/or upgrades for each outcome. DRM indicate dose response meta-analysis; NA, not applicable.

Figure 2 and eFigures 1 through 8 in the Supplement illustrate the association between food sources of fructose-containing sugars and incident MetS. Intake of SSBs was associated with an increased risk of incident MetS (RR, 1.20; 95% CI, 1.06-1.36), with evidence of significant heterogeneity^45^ (*I*^2^ = 68%; *P* = .005 determined by the *Q* statistic). Fruit and yogurt intake had an inverse association with incident MetS (fruit: RR, 0.91; 95% CI, 0.89-0.93; *I*^2^ = 0%; *P* = .78 determined by the *Q* statistic; yogurt: RR, 0.83; 95% CI, 0.77-0.90; *I*^2^ = 65%; *P* = .02 determined by the *Q* statistic). No association was found between mixed fruit juice, 100% fruit juice, honey, ice cream, or confectionary with MetS incidence.

### Sensitivity Analyses

eTable 5 in the Supplement details the sensitivity analysis after systematic removal of each cohort study for food sources with more than 2 studies. Results for SSBs did not alter in direction and significance of association (eg, maintained an adverse association with MetS incidence) or the evidence of heterogeneity. Similar results were found for mixed fruit juice and fruit, where removal of each study maintained no association for mixed fruit juice and a significant protective association for fruit. Heterogeneity in both mixed fruit juice and fruit remained nonsignificant. Removal of the study by Cheraghi et al^30^ resulted in nonsignificant evidence of interstudy heterogeneity for yogurt; however, it did not significantly affect the pooled estimate. Because none of the comparisons had 10 or more cohorts, subgroup analyses were not performed.

### Dose Response

**Figure 3.**
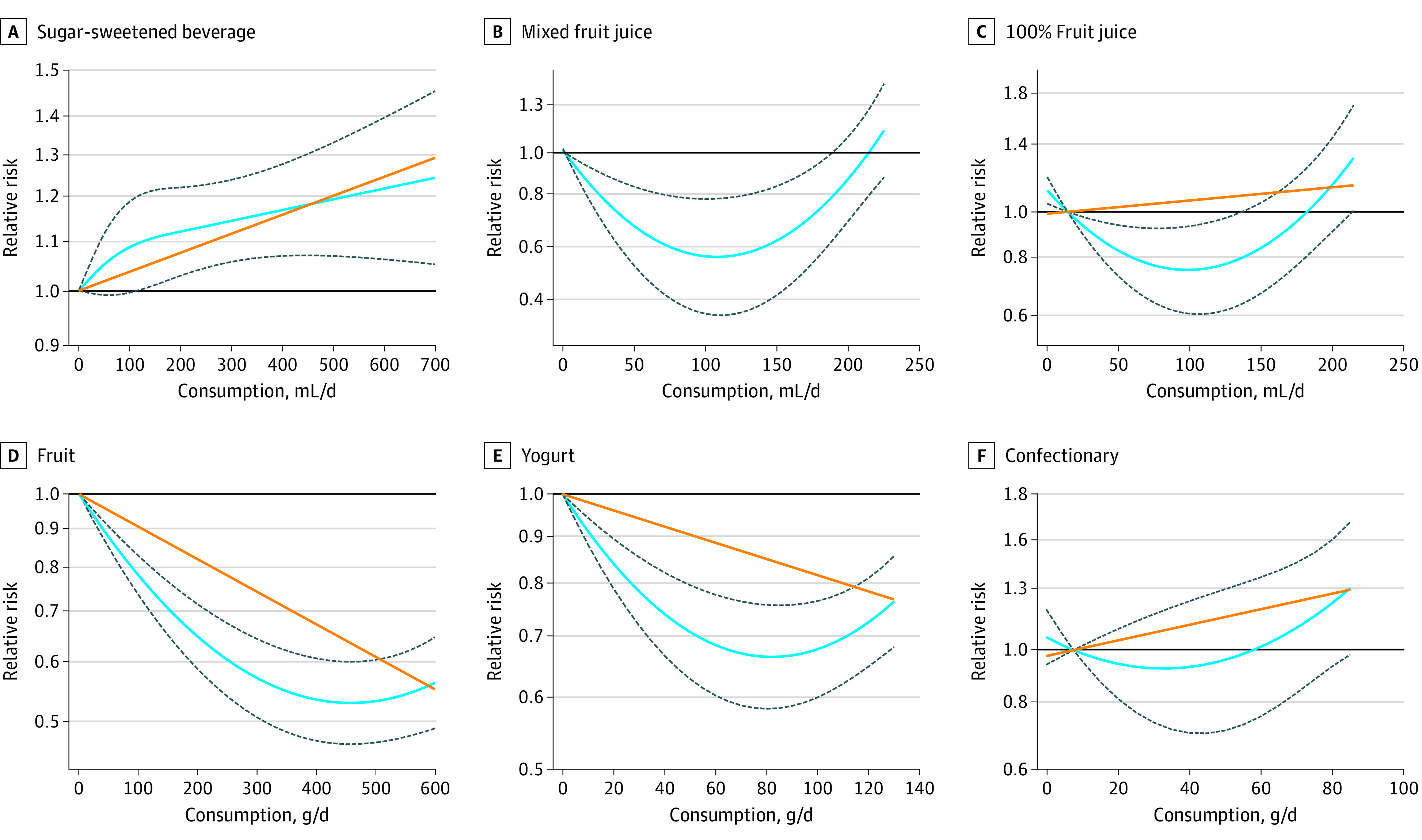
Dose-Response Association of Food Sources of Fructose-Containing Sugars and Incident Metabolic Syndrome Dose-response association between intake of sugar-sweetened beverages (linearity: risk ratio [RR] per 355 mL, 1.14; 95% CI, 1.05-1.23; *P* = .001; departure from linearity: RR per 355 mL, 1.16; 95% CI, 1.07-1.26; *P* = .27) (A), mixed fruit juice (linearity: RR per 125 mL, 1.00; 95% CI, 0.87-1.14; *P* = .96; departure from linearity: RR per 125 mL, 0.58; 95% CI, 0.42-0.79; *P* < .001) (B), 100% fruit juice (linearity: RR per 125 mL, 1.09; 95% CI, 0.93-1.27; *P* = .31; departure from linearity: RR per 125 mL, 0.77; 95% CI, 0.51-0.97; *P* < .001) (C), fruit (linearity: RR per 80 g, 0.92; 95% CI, 0.91-0.94; *P* < .001; departure from linearity: RR per 80 g, 0.82; 95% CI, 0.78-0.85; *P* < .001) (D), yogurt (linearity: RR per 85 g, 0.92; 95% CI, 0.91-0.94; *P* < .001; departure from linearity: RR per 85 g, 0.66; 95% CI, 0.58-0.76; *P* < .001) (E), and confectionary (linearity: RR per 50 g, 1.18; 95% CI, 0.98-1.42; *P* = .07; departure from linearity: RR per 50 g, 0.96; 95% CI, 0.71-1.30; *P* = .17) (F) with the risk of metabolic syndrome. The solid orange line represents the linear model and the blue line the nonlinear model. Dotted lines represent 95% CIs for the nonlinear model.

Figure 2 and Figure 3 show the dose-response association of each food source and incident MetS. Data from 6 cohorts,^32,34,36,37,38^ with a dose range of 0 to 680 mL/d, demonstrated an adverse linear dose-response association between SSB intake and MetS (RR for 355 mL/d, 1.14; 95% CI, 1.05-1.23), with no evidence for departure from linearity (*P* = .27) (Figure 3).

Data for mixed fruit juice (2 cohorts^32,38^) and 100% fruit juice (1 cohort^32^) indicate a U-shaped, significant, nonlinear dose-response association with incident MetS, with the curve suggesting a maximum protection between 75 and 150 mL. There was no protective association after 200 mL/d for mixed fruit juice intake and after 175 mL/d for 100% fruit juice. The estimated RR for 125 mL/d was 0.58 (95% CI, 0.42-0.79) for mixed fruit juice and 0.77 (95% CI, 0.61-0.97) for 100% fruit juice.

Data from 2 cohorts^40^ with a dose range of 0 to 600 g/d found a significant L-shaped, protective, nonlinear dose response for fruit intake and incident MetS, suggesting a sharp reduction of RR until 450 g/d. The estimated RR for 240 g (3 servings) was 0.61 (95% CI, 0.55-0.68).

Data from 3 cohorts^29,35^ with a dose range of 0 to 129 g/d of yogurt intake found an L-shaped, protective, nonlinear dose-response association with incident MetS, with the curve suggesting a sharp reduction of RR until 80 g/d. The estimated RR for 85 g (one-third cup serving) was 0.66 (95% CI, 0.58-0.76).

Confectionary data from 2 cohorts^37^ with a dose range of 8 to 84 g/d found no evidence of a dose-response association with incident MetS (RR per 50 g, 1.18; 95% CI, 0.98-1.42). Relevant data were not available to assess the dose-response association for honey and ice cream.

### GRADE Assessment

The GRADE certainty of evidence was moderate for adverse association for SSBs and protective association for mixed fruit juice, 100% fruit juice, fruit, and yogurt with MetS risk attributable to upgrades for dose-response gradient (Figure 2 and eTable 4 in the Supplement). Although both SSBs and 100% fruit juice had substantial interstudy heterogeneity (*I*^2^ = 68% for SSBs and 73% for fruit juice), the RR estimates for SSB studies were all in the same direction with considerable overlap. In addition, the heterogeneity observed with 100% fruit juice was explained by the nonlinear dose-response model. Therefore, these 2 food sources were not downgraded for inconsistency. The certainty of evidence of no association was very low for honey, ice cream, and confectionary because of downgrades for serious imprecision, indirectness for honey, ice cream, and confectionary with no upgrades.

## Discussion

In our systematic review and meta-analysis, 13 prospective cohort studies (including 49 591 participants and 14 205 MetS cases) found that SSB intake was associated with an increased risk for MetS incidence, whereas yogurt and fruit were associated with a reduced risk. Mixed fruit juice and 100% fruit juice had a U-shaped association with MetS, presenting a protective association between 75 and 150 mL/d and an adverse association for more than 175 to 200 mL/d. No association was found between honey, ice cream, and confectionary and MetS incidence.

The adverse association of SSB intake and MetS incidence in our study is consistent with the current literature.^6^ Previous meta-analyses^6,46^ found a 20% and 46% increased MetS risk with higher SSB consumption from 3 prospective and 8 cross-sectional studies, respectively. Our findings expand on current findings by the inclusion of 7 prospective cohorts and the assessment of dose response, which found a 14% increased risk of MetS incidence per 355-mL daily serving of SSBs.

The association between SSB and incident MetS may reflect a general unhealthy lifestyle whereby individuals with greater SSB intake are likely to have a poorer diet quality, higher caloric intake, and a sedentary lifestyle.^47^ Furthermore, SSBs are a source of liquid calories, which can have a lower effect on satiety compared with solid foods, resulting in increased energy intake, weight gain, and downstream complications related to MetS.^48^ Although the prospective studies^28,29,30,31,32,33,34,35,36,37,38,39,40^ included in our SSB analysis controlled for potential confounding factors, all except 1 study^28^ controlled for total energy intake, and 2 studies^28,36^ did not adjust for adiposity, an important risk factor and component of MetS.^3^ Thus, residual and unmeasured confounding could have contributed to the observed adverse association.

Conversely, yogurt had a protective association against MetS incidence, with a dose-dependent benefit with intakes of 60 to 80 g/d. The nonlinear findings indicate that the association above 85 g/d plateaus, and data are lacking to suggest any benefit associated with increasing intake beyond this dose. The role of yogurt, or more broadly dairy intake, and MetS has gained attention during the past decade. A meta-analysis^49^ highlighted that higher dairy consumption was inversely associated with MetS incidence by 14% among 7 prospective cohorts with a dose-response reduction with incremental intake. Our findings broadly concur with these results. This protective association of yogurt may be attributable to its rich micronutrient composition. Calcium, a major nutrient in yogurt, decreases fat absorption, lowers triglyceride concentration, improves the overall ratio of high-density lipoprotein to low-density lipoprotein.^50^ In addition, dairy-derived saturated fats have anti-inflammatory properties and potentially improve insulin sensitivity and glycemic response.^51^ Furthermore, the probiotic bacteria found in yogurt products have been linked to modulating gut microbiota through the reduction of pathogenic bacteria while increasing metabolite production and modulating various inflammatory reactions, all of which can aid in reducing the risk of MetS.^52^

Similarly, fruit consumption presented a protective association against MetS incidence, with the greatest dose benefit at 300 to 450 g/d (equivalent to 3-5 servings). Fruit intake is protective for some components of MetS, including waist circumference^53^ and blood pressure^54^; however, evidence on the dose range with MetS is limited.^40^ Most fruit intake and MetS studies^36,55,56^ are cross-sectional in design or are assessed in combination with vegetable intake, making it difficult to determine the association of specific fructose-containing fruits with MetS. One such meta-analysis^57^ of cross-sectional studies found that fruit intake had a protective association with MetS risk.

We identified a U-shaped dose-dependent association with mixed fruit juice and 100% fruit juice intake, showing protective associations against MetS with intakes less than 200 mL. The benefit of 100% fruit juice seen at moderate doses may be attributable to the range of fruit-derived nutrients and bioactive compounds in fruit juice,^58^ and the potential for harm at higher doses may be attributable to the consumption of excess calories outweighing any benefit of these bioactive nutrients.^59^

Mixed fruit juices are a combination of fruit drinks (which are similar to SSBs because they are sugary drinks without the accompanying nutrients) and 100% fruit juice. The observed moderate doses of intake may represent the beneficial nutrients from natural fruit within the mixed fruit juice, thus indicating an association similar to that of 100% fruit juices rather than SSBs. The lack of linear association in 100% fruit juice and mixed fruit juice underscores that without consideration of the dose-response association, a naive analysis of extreme intakes assumes a false-linear association and fails to detect important dose ranges for protection or harm.^47^

Furthermore, honey, ice cream, and confectionary intake was not associated with MetS incidence. Although animal models suggest potential protective effects of honey in MetS,^60^ to our knowledge, only 1 prospective cohort study^30^ assessed honey with MetS incidence and found no significant association. Similarly, the current limited evidence indicates that ice cream^30^ and confectionary^37^ were not significantly associated with MetS incidence. Future data might clarify our association, particularly for confectionaries, for which CIs did not eliminate significant harm.

The protective and neutral association in our results highlight 2 important considerations. First, the small beneficial effects of some foods might be driven by catalytic doses of fructose intake. Second, the food composition is important. SSBs are without beneficial nutrients and thus offer an unchecked source of fructose-containing sugar, whereas in other foods (eg, yogurt), nutrients other than sugars (eg, polyphenols, minerals, and fiber) may offer protection that might overcome harms from added sugars. More data are needed to enable a complete dose-response assessment and reveal dose ranges for increased or reduced risk, depending on the balance between nutrient matrixes vs excess sugars.

### Strengths and Limitations

There are numerous strengths associated with our study. To our knowledge, this study is the first meta-analysis to comprehensively compare major food sources of fructose-containing sugars with incident MetS in prospective cohort studies. We conducted a thorough literature search, performed quantitative synthesis, and assessed the certainty of the evidence using GRADE. Selected studies included a large sample size, long follow-up durations, and adjustment for multiple lifestyle factors. We also assessed dose responses for all food sources and identified ranges and cutoffs for benefit and harm.

This study also had some limitations. The observational nature of prospective cohort studies may result in unmeasured and residual confounding and may suffer from reverse causality. Thus, GRADE evaluation for observational studies is low certainty of evidence. Although SSBs, yogurt, and 100% fruit juice had substantial interstudy heterogeneity, we did not consider this as a serious inconsistency.^61^ The estimates were all in the same direction, and there was considerable overlap for SSB and yogurt. The nonlinear dose-response model explained the heterogeneity for yogurt and 100% fruit juice. Honey, ice cream, and confectionary were downgraded for serious indirectness for the inability to assess inconsistency because only 1 study was available for each exposure. Furthermore, they were downgraded for serious imprecision, indicating no association with MetS incidence in the extreme quantile analysis. The CIs were wide and could not conclude clinically important harm for confectionary or clinically important benefit or harm for honey and ice cream. In our dose-response analysis, we found a significant linear dose response of harm for SSBs and a nonlinear dose response of benefit for mixed fruit juice, 100% fruit juice, fruit, and yogurt, leading to an upgrade for the certainty of evidence. Data were not available for grain and grain-based products, a leading source of sugar.^62^

## Conclusions

Our study provides supporting evidence that increased SSB consumption is associated with MetS incidence. Generalizing statements on the adverse effects of fructose-containing sugars, however, cannot be extrapolated to other major food sources of fructose-containing sugars. Furthermore, our dose-response assessment found that mixed and 100% fruit juice presented consistent dosage for benefit that align with some national nutrition guidelines, suggesting that a 150-mL intake may contribute toward the recommended daily fruit consumption.^63,64^ Thus, well-intentioned policies and guidelines to limit sources of free sugars, such as fruit juice or sweetened yogurts, based on evidence from SSBs may need to be reexamined with a food-based lens, such as those of the new Canada’s Food Guide^65^ or Scientific Advisory Committee on Nutrition.^66^

Additional prospective studies are needed to improve our estimates and better understand the dose-response association between important food sources of fructose-containing sugars and MetS. Moreover, high-quality, large randomized clinical trials are needed on other fructose-containing foods. Furthermore, studies of whole diets and dietary patterns that consist of various food sources of fructose-containing sugars with cardiometabolic-related health outcomes can also contribute to the evidence of the association of these diets with MetS.

## Notes

Supplement. **eAppendix 1.** Details on GRADE **eAppendix 2.** Conversion of OR to RR **eAppendix 3.** Method for Dose-Response Analysis **eAppendix 4.** Definition of MetS **eTable 1.** Search Strategy **eTable 2.** Analysis of Confounding Variables Among 13 Studies of Food Sources of Fructose-Containing Sugars and Incident MetS **eTable 3.** Newcastle-Ottawa Scale (NOS) for Assessing the Quality of Cohort Studies **eTable 4.** GRADE Assessment **eTable 5.** GRADE Assessment (Continued) **eFigure 1.** Relationship Between SSB Intake and Incident MetS **eFigure 2.** Relationship Between Mixed Fruit Juice Intake and Incident MetS **eFigure 3.** Relationship Between 100% Fruit Juice Intake and Incident MetS **eFigure 4.** Relationship Between Fruit Intake and Incident MetS **eFigure 5.** Relationship Between Yogurt Intake and Incident MetS **eFigure 6.** Relationship Between Honey Intake and Incident MetS **eFigure 7.** Relationship Between Ice-Cream Intake and Incident MetS **eFigure 8.** Relationship Between Confectionary Intake (Including Cakes, Biscuits, Chocolate and Candies) and Incident MetS **eReferences**
