# Supplementary material for: Association of Major Food Sources of Fructose-Containing Sugars With Incident Metabolic Syndrome: A Systematic Review and Meta-analysis
Source: JAMA Netw Open. 2020 Jul 9;3(7):e209993. doi: 10.1001/jamanetworkopen.2020.9993 (PMC7348689; doi:10.1001/jamanetworkopen.2020.9993)
Supplement: Supplement. — eAppendix 1. Details on GRADE eAppendix 2. Conversion of OR to RR eAppendix 3. Method for Dose-Response Analysis eAppendix 4. Definition of MetS eTable 1. Search Strategy eTable 2. Analysis of Confounding Variables Among 13 Studies of Food Sources of Fructose-Containing Sugars and Incident MetS eTable 3. Newcastle-Ottawa Scale (NOS) for Assessing the Quality of Cohort Studies eTable 4. GRADE Assessment eTable 5. GRADE Assessment (Continued) eFigure 1. Relationship Between SSB Intake and Incident MetS eFigure 2. Relationship Between Mixed Fruit Juice Intake and Incident MetS eFigure 3. Relationship Between 100% Fruit Juice Intake and Incident MetS eFigure 4. Relationship Between Fruit Intake and Incident MetS eFigure 5. Relationship Between Yogurt Intake and Incident MetS eFigure 6. Relationship Between Honey Intake and Incident MetS eFigure 7. Relationship Between Ice-Cream Intake and Incident MetS eFigure 8. Relationship Between Confectionary Intake (Including Cakes, Biscuits, Chocolate and Candies) and Incident MetS eReferences [file jamanetwopen-3-e209993-s001.pdf]

Supplementary Online Content

Semnani-Azad Z, Khan TA, Blanco Mejia S, et al. Association of major food sources of fructose-containing sugars with incident metabolic syndrome: a systematic review and meta-analysis. *JAMA Netw Open*. 2020;3(7):e209993. doi:10.1001/jamanetworkopen.2020.9993

eAppendix 1. Details on GRADE

eAppendix 2. Conversion of OR to RR

eAppendix 3. Method for Dose-Response Analysis

eAppendix 4. Definition of MetS

eTable 1. Search Strategy

eTable 2. Analysis of Confounding Variables Among 13 Studies of Food Sources of Fructose-Containing Sugars and Incident MetS

eTable 3. Newcastle-Ottawa Scale (NOS) for Assessing the Quality of Cohort Studies

eTable 4. GRADE Assessment

eTable 5. GRADE Assessment (Continued)

eFigure 1. Relationship Between SSB Intake and Incident MetS

eFigure 2. Relationship Between Mixed Fruit Juice Intake and Incident MetS

eFigure 3. Relationship Between 100% Fruit Juice Intake and Incident MetS

eFigure 4. Relationship Between Fruit Intake and Incident MetS

eFigure 5. Relationship Between Yogurt Intake and Incident MetS

eFigure 6. Relationship Between Honey Intake and Incident MetS

eFigure 7. Relationship Between Ice-Cream Intake and Incident MetS

eFigure 8. Relationship Between Confectionary Intake (Including Cakes, Biscuits, Chocolate and Candies) and Incident MetS

eReferences

This supplementary material has been provided by the authors to give readers additional information about their work.

© 2020 Semnani-Azad et al. *JAMA Network Open*.

eAppendix 1. Details on GRADE

GRADE (Grading of Recommendations Assessment, Development and Evaluation) was used to evaluate the quality of each study. Evidence extracted from observational studies were defaulted to 'low'- certainty and were downgraded or upgraded based on pre-specified criteria. Criteria to downgrade included risk of bias (weight of studies show risk of bias as assessed by NOS<6), inconsistency (substantial unexplained inter-study heterogeneity I^2>50%, P_Q<0.10), indirectness (presence of factors that limit the generalizability of the results), imprecision in the pooled risk estimate (the 95% CI for risk estimates that cross a minimally important difference of 5% for benefit or harm [RR 0.95–1.05]), and publication bias (evidence of small-study effects). Upgraded criteria included a large magnitude of effect (RR>2 or RR<0.5 in the absence of plausible confounders), dose–response gradient, and attenuation of the pooled effect estimate by plausible confounders.

© 2020 Semnani-Azad et al. *JAMA Network Open*.

eAppendix 2. Conversion of OR to RR

For studies with reported hazard ratios, low incidence of MetS (<10%) or odds ratios (OR) between 0.5 and 2.5, values were treated as RRs. OR were converted to RR if OR was less than 0.5 or greater than 2.5 with an incident of MetS greater than 10%. As outlined by Zhang et al.[1], the following formulae and logic were applied:

$$OR = \frac{\left(\frac{P_1}{1 - P_1}\right)}{\left(\frac{P_0}{1 - P_0}\right)}$$

Thus,

$$\frac{P_1}{P_0} = \frac{OR}{[(1 - P_0) + (P_0 \; x \; OR)\,]}$$

RR = $\frac{P_1}{P_0}$, therefore:

$$RR = \frac{OR}{[(1 - P_0) + (P_0 \; x \; OR)\,]}$$

P_0 = incidence of the outcome of interest in the non-exposed group; P_1 = incidence of the outcome of interest in the exposed group.

© 2020 Semnani-Azad et al. *JAMA Network Open*.

eAppendix 3. Method for Dose-Response Analysis

We modelled dose-response model using RR and 95% CIs from dose categories to understand the shape of the association between the dose of the food source of fructose-containing sugar and the risk of MetS. Data on the dose, distribution of cases and person-years, RRs and 95% CIs were extracted from each study. We defined the assigned dose as the mean consumption in each reported category or quantile. If the assigned were not reported, we approximated the mean dose for each category by using the midpoint of its lower and upper bounds. If the lowest category of a study was open ended, we defined the lowest dose as zero. For open-ended upper categories, we took half of the adjacent category range to estimate the assigned dose. When cohort size or person-year per category were not available, categories were regarded equal in size and follow-up and the case number per category was obtained by the method of Bekkering[2]. We excluded studies from the dose–response meta-analysis that did not report any dose category cut points for the particular food source and studies that provided only RR estimates based on 1-unit increment in dose based on a linear model because these studies were unable to contribute to the assessment of departure from linearity. We fitted a dose-response relationship using restricted cubic splines with 3 knots at 15th, 50th and 85th percentiles of distribution taking into account the correlation within each category of published RRs and combining the study-specific estimates the one-stage linear mixed-effects meta-analysis [3]. This method estimates the study specific slope lines and combines them to obtain an overall average slope based upon the work of Greenland [4] and Orsini [5]. If restricted cubic splines could not be calculated due to limited number of observations, we fitted a second order fractional polynomial curve to the data [5] and tested for goodness-of-fit of the model using Akaike information criterion (AIC), deviance test (D) and the coefficient of determination (R2) to select the best-fitting model [6]. We reported non-linear associations for a study if Wald test for departure from linearity was significant at $p<0.10$. RRs below 1 were considered as protective and above 1 as adverse association.

© 2020 Semnani-Azad et al. *JAMA Network Open*.

eAppendix 4. Definition of MetS

The harmonized criteria classification for MetS takes into account definitions set by the International Diabetes Federation (IDF) and the American Heart Association/ National Heart, Lung, and Blood Institute ATPIII.[7] Both IDF and ATP III definitions include thresholds for waist circumference, elevated triglycerides of ≥150 mg/dL, low HDL of <40 mg/dL in males and <50 mg/ in females, elevated blood pressure of systolic ≥130 and/or diastolic ≥85 mm Hg, and elevated fasting blood glucose of ≥150 mg/d.[8] The ATP III identifies MetS as the presence of any 3 of 5 risk factors. The IDF defines MetS as the presence of abdominal obesity measured through waist circumference, with the addition of any 2 of 4 risk factors. The harmonized criteria defines MetS as the presence of any 3 of 5 risk factors, with specific waist circumference cut-points depending on ethnicity.[8]

© 2020 Semnani-Azad et al. *JAMA Network Open*.

eTable 1. Search Strategy.

MEDLINE		EMBASE		Cochrane	
1	sugar*.mp.	1	sugar*.mp.	1	sugar*.mp.
2	exp fructose/	2	exp sugar/	2	exp fructose/
3	fructose.mp.	3	exp fructose/	3	fructose.mp.
4	HFCS.mp.	4	fructose.mp.	4	HFCS.mp.
5	exp High Fructose Corn Syrup/	5	HFCS.mp.	5	exp Nutritive Sweeteners/
6	sucrose.mp.	6	exp high fructose corn syrup/	6	sucrose.mp.
7	exp Dietary Sucrose/	7	sucrose.mp.	7	exp dietary sucrose/
8	sugar sweetened beverage*.mp.	8	exp dietary sucrose/	8	sugar sweetened beverage*.mp.
9	SSB.mp.	9	sugar sweetened beverage*.mp.	9	ssb.mp.
10	soda.mp.	10	SSB.mp.	10	soda.mp.
11	soft drink*.mp.	11	soda.mp.	11	soft drink*.mp.
12	exp Carbonated Beverages/	12	soft drink*.mp.	12	exp carbonated beverages/
13	carbonated beverages.mp.	13	exp soft drink/	13	non alcoholic beverage*.mp.
14	non alcoholic beverage*.mp.	14	exp Carbonated Beverages/	14	nonalcoholic beverage*.mp.
15	nonalcoholic beverage*.mp.	15	carbonated beverages.mp.	15	exp energy drinks/
16	exp Energy Drinks/	16	non alcoholic beverage*.mp.	16	energy drink*.mp.
17	energy drink*.mp.	17	nonalcoholic beverage*.mp.	17	smoothie*.mp.
18	smoothie*.mp.	18	exp energy drink/	18	((fruit or vegetable) and juice*).mp.
19	exp "Fruit and Vegetable Juices"/	19	energy drink*.mp.	19	fruit.mp.
20	fruit.mp.	20	smoothie*.mp.	20	exp fruit/
21	exp Fruit/	21	exp "fruit and vegetable juice"/	21	exp honey/
22	exp Honey/	22	fruit.mp.	22	y*g*rt.mp.
23	y*g*rt.mp.	23	exp fruit/	23	exp yogurt/
24	exp Yogurt/	24	exp honey/	24	ice cream*.mp.
25	ice cream*.mp.	25	y*g*rt.mp.	25	icecream*.mp.
26	icecream*.mp.	26	exp yoghurt/	26	exp ice cream/
27	exp Ice Cream/	27	exp ice cream/	27	cereal*.mp.
28	cereal*.mp.	28	ice cream*.mp.	28	dessert*.mp.
29	exp edible grain/	29	icecream*.mp.	29	sweets.mp.
30	dessert*.mp.	30	cereal*.mp.	30	confection*.mp.
31	sweets.mp.	31	dessert*.mp.	31	pastries.mp.
32	confection*.mp.	32	sweets.mp.	32	biscuit*.mp.
33	pastries.mp.	33	confection*.mp.	33	cookie*.mp.
34	biscuit*.mp.	34	exp bakery product/	34	cake*.mp.
35	cookie*.mp.	35	pastries.mp.	35	candy.mp.

© 2020 Semnani-Azad et al. *JAMA Network Open*.

36	cake*.mp.	36	biscuit*.mp.	36	candies.mp.
37	candy.mp.	37	cookie*.mp.	37	exp candy/
38	candies.mp.	38	cake*.mp.	38	(chocolate adj2 milk).mp.
39	exp Candy/	39	candy.mp.	39	exp chocolate/
40	(chocolate adj2 milk).mp.	40	candies.mp.	40	Chocolate.mp
41	exp chocolate/	41	(chocolate adj2 milk).mp.	41	exp cacao/
42	Chocolate.mp	42	exp chocolate/	42	cacao.mp.
43	exp cacao/	43	Chocolate.mp	43	or/1-42
44	cacao.mp.	44	exp cacao/	44	cohort.mp.
45	or/1-44	45	cacao.mp.	45	exp Prospective Studies/
46	cohort.mp.	46	or/1-45	46	(prospective adj2 (cohort or study)).mp.
47	exp prospective study/	47	cohort.mp.	47	exp follow-up studies/
48	(prospective adj2 (cohort or study)).mp.	48	exp prospective study/	48	exp multivariate analysis/
49	exp Follow-Up Studies/	49	(prospective adj2 (cohort or study)).mp.	49	exp proportional hazards models/
50	exp Multivariate Analysis/	50	exp multivariate analysis/	50	follow up study.mp.
51	exp Proportional Hazards Models/	51	exp proportional hazards model/	51	(longitudinal adj2 study).mp.
52	follow up study.mp.	52	follow up study.mp.	52	or/44-51
53	(longitudinal adj2 study).mp.	53	(longitudinal adj2 study).mp.	53	metabolic syndrome.mp.
54	or/46-53	54	or/47-53	54	syndrome x.mp.
55	metabolic syndrome.mp.	55	metabolic syndrome.mp.	55	cardio-metabolic syndrome.mp.
56	syndrome x.mp.	56	syndrome x.mp.	56	MetS.mp.
57	cardio-metabolic syndrome.mp.	57	cardio-metabolic syndrome.mp.	57	or/53-56
58	MetS.mp.	58	MetS.mp.	58	43 and 52 and 57
59	or/55-58	59	or/55-58		
60	45 and 54 and 59	60	46 and 55 and 59		

**Database**	**Total**
MEDLINE: March week 3, 2020	402
EMBASE: March week 3, 2020	584
Cochrane: March week 3, 2020	76
Manual search	8
**Total**	**1071**
**Duplicates**	**396**
**Final Total**	**675**

© 2020 Semnani-Azad et al. *JAMA Network Open*.

eTable 2. Analysis of confounding variables among 13 studies of food sources of fructose-containing sugars and incident MetS.

Study	Appelhans et al., 2017 – SWAN[9]	Babio et al., 2015 - PREDIMED[10]	Cheraghi et al., 2016 – TLGS[11]	Duffey et al., 2010 – CARDIA[12]	Ferreira-Pego et al., 2016 – PREDIMED[13]	Hur et al., 2016 – KoCAS[14]	Kang and Kim, 2017 – KoGES[15]	Kim and Kim, 2017 – KoGES[16]	Lim and Kim , 2019 - KoGES[17]	Lutsey et al., 2008 - ARIC[18]	Mirmiran et al., 2014 – TLGS[19]	Mirmiran et al., 2015 – TLGS[20]	Sayon-Orea et al., 2015 – SUN[21]
**Number of variables in fully adjusted model**	10	23	0	10	26	4	14	12	14	14	15	12	15
**PRESPECIFIED VARIABLES**													
Age	✓	✓		✓	✓	✓	✓	✓	✓	✓	✓	✓	✓
Sex		✓		✓	✓	✓				✓	✓	✓	✓
Markers of overweight/ obesity (body mass index, weight, waist circumference, waist to hip ratio)		✓		✓	✓		✓	✓	✓		✓	✓	✓
Smoking	✓	✓		✓	✓		✓	✓	✓	✓	✓		✓
Family history of MetS								✓				✓	
Energy or caloric intake				✓	✓	✓	✓	✓	✓	✓	✓	✓	✓
Diabetes							✓						
Physical activity	✓	✓		✓	✓		✓	✓	✓	✓	✓	✓	✓
Alcohol		✓		✓	✓		✓	✓	✓				✓
**OTHER COVARIATE VARIABLES**													
Ethnicity	✓			✓						✓			
Education	✓						✓	✓	✓	✓	✓		
Hypertension/SBP		✓			✓								
HDL cholesterol		✓			✓								
Vegetables		✓			✓				✓	✓	✓	✓	
Fruit		✓			✓					✓	✓	✓	
Whole grain									✓	✓			
Fibre							✓	✓		✓	✓	✓	
Saturated fat							✓						
Unsaturated fat							✓						
Red meat		✓			✓				✓	✓		✓	✓
Coffee												✓	
Meat and fish		✓			✓								
Anti-hypertensive medication		✓			✓								
HRT		✓			✓								
Menopause	✓												

© 2020 Semnani-Azad et al. *JAMA Network Open*.

Insulin		✓			✓								
**OTHER**													
Hormone therapy use	✓												
Depressive symptoms	✓												
Income	✓					✓	✓	✓	✓				
Study site	✓			✓					✓				
Hypoglycemia and hypolipidemic drug		✓											
Legume		✓			✓								
Cereal		✓			✓								
Baked goods		✓			✓								
Nuts		✓			✓								
Olive oil		✓			✓								
High fasting plasma glucose		✓			✓								
Hypertriglycermidemia		✓			✓								
Trans fat													
Glycemic index													
Magnesium													
Dairy products					✓				✓	✓			
Percentage of fat							✓						
Presence of disease							✓						
Income													
Residential location								✓					
Calcium								✓					
Weight change											✓		
Phytochemical index											✓		
Dietary total antioxidant capacity											✓		
Tea												✓	
French fries													✓
Fast food													✓
Mediterranean diet													✓
Sedentary behaviour													
Hours sitting													✓
Snacking between meals													✓
Special diet													✓
Refined grains									✓	✓			

✓ Means variable adjusted for in the most adjusted model.

© 2020 Semnani-Azad et al. *JAMA Network Open*.

eTable 3. Newcastle-Ottawa Scale (NOS) for assessing the quality of cohort studies.

Study, year	Selection*	Outcome†	Comparability‡	Total§
Appelhans et al. 2017[9]	4	2	2	8
Babio et al. 2015[10]	3	2	2	7
Cheraghi et al. 2016[11]	4	2	0	6
Duffey et al. 2010[12]	4	2	2	8
Ferreira-Pego et al. 2016[13]	3	3	2	8
Hur et al. 2016[14]	4	2	1	6
Kang and Kim 2017[15]	4	2	2	8
Kim and Kim 2017[16]	4	2	2	8
Lim and Kim 2019 [17]	4	2	2	8
Lutsey et al. 2008[18]	3	3	2	8
Mirmiran et al. 2014[19]	3	2	2	7
Mirmiran et al. 2015[20]	3	2	2	7
Sayon-Orea et al. 2015[21]	3	3	2	8

*Maximum 4 points awarded for cohort representativeness, selection of non-exposed cohort, exposure assessment, and demonstration outcome not present at baseline

†Maximum 3 points awarded for follow-up length, adequacy of follow-up, and outcome assessment

‡ Maximum 2 points awarded for controlling for the pre-specified primary confounding variable (age) and additional confounding variables.

§ A maximum of 9 points could be awarded

© 2020 Semnani-Azad et al. *JAMA Network Open*.

eTable 4. GRADE Assessment.

Quality assessment								Study event rates (%)	Estimate	Quality Importance
**No. of studies**	**Design**	**Risk of bias**	**Inconsistency**	**Indirectness**	**Imprecision**	**Publication bias**	**Other consideration**		**Relative Risk (95% CI)**	
**SSB intake on incident MetS (follow-up mean 7.5 years)**										
7[9,13,15,18-20]	Observational Studies	Not serious	Not serious*	Not serious	Not serious[≠]	Undetected[±]	Dose-response association[ϴ]	7,406/20,480 (36%)	1.21 [1.06-1.37]<br><br>**Linear DRM**<br>$RR_{355\text{-ml/day}}$<br>1.14 [1.05, 1.23]	⊕⊕⊕⊖<br>**MODERATE**[*,≠,±,ϴ]<br>Upgrade due to dose-response association.
**Mixed fruit juice intake on incident MetS (follow-up mean 3.4 years)**										
3[13,14,20]	Observational Studies	Not serious	Not serious	Not serious	Not serious[≠≠]	Undetected[±]	Dose-response association [ϴϴ]	1,322/3,062 (43%)	1.13 [0.91-1.41]<br><br>**Non-linear DRM**<br>$RR_{125\text{-ml/day}}$<br>0.58 [0.42, 0.79]	⊕⊕⊕⊖<br>**MODERATE**[≠≠, ±, ϴϴ]<br>Upgrade for dose-response association.
**100% Fruit juice intake on incident MetS (follow-up mean 5.1 years)**										
2[12,13]	Observational Studies	Not serious	Not serious**	Not serious	Not serious[≠≠≠]	Undetected[±]	Dose-response association [ϴϴϴ]	1,389/5,464 (25%)	1.10 [0.84-1.44]<br><br>**Non-linear DRM**<br>$RR_{125\text{-ml/day}}$<br>0.77 [0.61, 0.97]	⊕⊕⊕⊖<br>**MODERATE****[, ≠≠≠, ±, ϴϴϴ]<br>Upgrade for dose-response association.
**Fruit on incident MetS (follow-up mean 4.7 years)**										
4[11,14,17]	Observational Studies	Not serious	Not serious	Not serious	Not serious[≠≠≠≠]	Undetected[±]	Dose-response association [ϴϴϴϴ]	3,002/10,074 (30%)	0.91 [0.89, 0.93]<br><br>**Non-linear DRM**<br>$RR_{80\text{gl/day}}$<br>0.82 [0.78, 0.86]	⊕⊕⊕⊖<br>**MODERATE**[≠≠≠≠, ±,ϴϴϴϴ]<br>Upgrade for dose-response association.
**Yogurt intake on incident MetS (follow-up mean 3.4 years)**										

© 2020 Semnani-Azad et al. *JAMA Network Open*.

5[10,11,16,21]	Observational Studies	Not serious	Not serious***	Not serious	Not serious####	Undetected±	Dose-response association ϴϴϴϴϴ	3,877/19,057 (20%)	0.83 [0.77, 0.90]<br><br>**Non-linear DRM**<br>$RR_{-85\text{-}g/day}$ 0.66 [0.58, 0.76]	⊕⊕⊕⊖ **MODERATE*****,####, ±, ϴϴϴϴϴ<br>Due to an upgrade for dose-response association.

© 2020 Semnani-Azad et al. *JAMA Network Open*.

eTable 4. GRADE Assessment *(Continued)*.

Quality assessment								Study event rates (%)	Estimate	Quality Importance
No. of studies	Design	Risk of bias	Inconsistency	Indirectness	Imprecision	Publication bias	Other consideration		Relative Risk (95% CI)	
**Honey intake on incident MetS (follow-up 2.05 years)**										
1[11]	Observational Studies	Not serious	Undetected****	Serious Ŧ	Serious≠≠≠≠≠	Undetected±	None	590/3,616 (16%)	1.00 [0.5, 2.00]	⊕⊖⊖⊖ **VERY LOW****,** Ŧ,≠≠≠≠≠,± Due to downgrade for serious indirectness and serious imprecision.
**Ice-cream intake on incident MetS (follow-up 2.05 years)**										
1[11]	Observational Studies	Not serious	Undetected****	Serious Ŧ	Serious≠≠≠≠≠≠	Undetected±	None	590/3,616 (16%)	0.94 [0.84, 1.06]	⊕⊖⊖⊖ **VERY LOW****,** Ŧ,≠≠≠≠≠≠,± Due to downgrade for serious indirectness and serious imprecision.
**Confectionary intake on incident MetS (follow-up 3 years)**										
2[19]	Observational Studies	Not serious	Not serious	Serious Ŧ	Serious≠≠≠≠≠≠≠	Undetected±	None	250/1,476 (17%)	1.21 [0.92, 1.60]	⊕⊖⊖⊖ **VERY LOW** Ŧ,≠≠≠≠≠≠≠,± Due to downgrade for serious indirectness and serious imprecision.

* Although there was evidence of substantial inter-study heterogeneity (I^2 = 68%), the estimates were all in the same direction and there was considerable overlap. Therefore, we did not consider this as serious inconsistency.

** There was substantial heterogeneity (I^2 = 73%, P_Q= 0.05) in the pairwise analysis. This was explained by the non-linear dose-response model. Therefore, we did not downgrade for serious inconsistency.

© 2020 Semnani-Azad et al. *JAMA Network Open*.

***	Although there was evidence of substantial inter-study heterogeneity (I^2 = 65%), the estimates were all in the same direction and there was considerable overlap. Therefore, we did not consider this as serious inconsistency.
****	Not able to assess inconsistency due to only one study included.
Ŧ	Downgrade for serious indirectness due to only one cohort available, therefore affecting the generalizability to the general population.
≠	No downgrade for serious imprecision as lower bound of 95% CI does not cross the clinically unimportant effects (RR 0.95 – 1.05).
≠≠	The potential imprecision from pairwise meta-analysis was explained by the non-linear dose-response model. Therefore, we did not downgrade for serious imprecision for mixed fruit juice.
≠≠≠	The potential imprecision from pairwise meta-analysis was explained by the non-linear dose-response model. Therefore, we did not downgrade for serious imprecision for 100% fruit juice.
≠≠≠≠	No downgrade for serious imprecision as lower bound of 95% CI does not cross the clinically unimportant effects (RR 0.95 – 1.05).
≠≠≠≠≠	No downgrade for serious imprecision as the upper bound of 95% CI (RR 0.90) does not include the threshold for clinically unimportant effects (RR 0.95 – 1.05).
≠≠≠≠≠≠	Downgrade for serious imprecision as the lower bound of 95% CI (RR 0.50) includes clinically important benefit (RR <0.90) while the upper bound of the 95% CI (RR 2.00) includes the clinically important harm (RR >1.05).
≠≠≠≠≠≠≠	Downgrade for serious imprecision as the lower bound of 95% CI (RR 0.84) includes clinically important benefit (RR <0.90) while the upper bound of the 95% CI (RR 1.06) includes the unimportant effects (RR 0.95 – 1.05).
≠≠≠≠≠≠≠≠	Downgrade for serious imprecision as the lower bound of 95% CI (RR, 0.92) includes clinically unimportant effects (RR 0.95 – 1.05) while the upper bound of the 95% CI (RR, 1.60) includes the clinically important harm (RR >1.05).
±	No downgrade for publication bias, as publication bias could not be assessed due to lack of power for assessing funnel plot asymmetry and small study effects (<10 cohorts included in our meta-analysis).
ϴ	Linear dose response relationship with suggestion of positive association with risk SSB (P=0.001).
ϴϴ	Non-linear dose response relationship with suggestion of inverse association with risk for mixed fruit juice (P<0.001).
ϴϴϴ	Non-linear dose response relationship with suggestion of inverse association with risk for 100% fruit juice (P<0.01).
ϴϴϴϴ	Linear and non-linear dose response relationship with suggestion of inverse association with risk for fruit (both P<0.001).
ϴϴϴϴϴ	Linear and non-linear dose response relationship with suggestion of inverse association with risk for yogurt (both P<0.001).
	DRM: Dose-response meta-analysis.

© 2020 Semnani-Azad et al. *JAMA Network Open*.

eTable 5. Sensitivity analysis for all food sources with more than 2 studies.

Removed Study	RR [95% CI]	P-value	Heterogeneity	
			I^2	P_Q
**SSB**				
All Studies Included	*1.21 [1.06, 1.37]*	*0.005*	*68%*	*0.005*
Appelhans, J Acad Nutr Diet, 2017 – SWAN[9]	1.37 [1.09, 1.71]	0.006	64%	0.016
Ferreira-Pêgo, J Nutri, 2016 – PREDIMED[13]	1.18 [1.04, 1.35]	0.013	69%	0.006
Kang, Br J Nutr, 2017 – KoGES (Female)[15]	1.13 [1.02, 1.26]	0.025	55%	0.048
Kang, Br J Nutr, 2017 – KoGES (Male) [15]	1.23 [1.07, 1.42]	0.005	73%	0.002
Lutsey, Circulation, 2008 – ARIC[18]	1.36 [1.07, 1.74]	0.012	73%	0.002
Mirmiran, Nutr Metab, 2015 – TLGS[20]	1.16 [1.03, 1.30]	0.011	62%	0.022
Mirmiran, Nutrition, 2014 – TLGS[19]	1.18 [1.04, 1.35]	0.013	69%	0.006
**Mixed Fruit Juice**				
All Studies Included	*1.13 [0.91, 1.43]*	*0.270*	*0%*	*0.867*
Ferreira-Pêgo, J Nutri, 2016 – PREDIMED[13]	1.04 [0.45, 2.40]	0.927	0%	0.622
Hur, Nutrients, 2015 - KoCAS[14]	1.14 [0.91, 1.43]	0.243	0%	0.934
Mirmiran, Nutr Metab, 2015 – TLGS[20]	1.13 [0.90, 1.42]	0.292	0%	0.599
**FRUIT**				
All Studies Included	*0.91 [0.89, 0.93]*	*<0.001*	*0%*	*0.778*
Cheraghi, Public Health, 2016 – TLGS[11]	0.91 [0.89, 0.93]	<0.001	0%	0.919
Hur, Nutrients, 2015 - KoCAS[14]	0.91 [0.89, 0.93]	<0.001	0%	0.628
Lim, Eur J Nutr, 2019 – KoGES (Female)[17]	0.91 [0.88, 0.95]	<0.001	0%	0.583
Lim, Eur J Nutr, 2019 – KoGES (Male)[17]	0.91 [0.88, 0.94]	<0.001	0%	0.580
**YOGURT**				
All Studies Included	*0.83 [0.77, 0.90]*	*<0.001*	*65%*	*0.021*
Babio, J Nutr, 2015 – PREDIMED[10]	0.85 [0.78, 0.92]	<0.001	72%	0.014
Cheraghi, Public Health, 2016 – TLGS[11]	0.74 [0.66, 0.82]	<0.001	0%	0.654
Kim, Br J Nutr, 2017 – KoGES (Female)[16]	0.85 [0.79, 0.93]	<0.001	66%	0.032
Kim, Br J Nutr, 2017 – KoGES (Male)[16]	0.85 [0.79, 0.93]	<0.001	61%	0.051
Sayon-Orea, BMC Public Health, 2015 – SUN[21]	0.83 [0.77, 0.90]	<0.001	74%	0.009

© 2020 Semnani-Azad et al. *JAMA Network Open*.

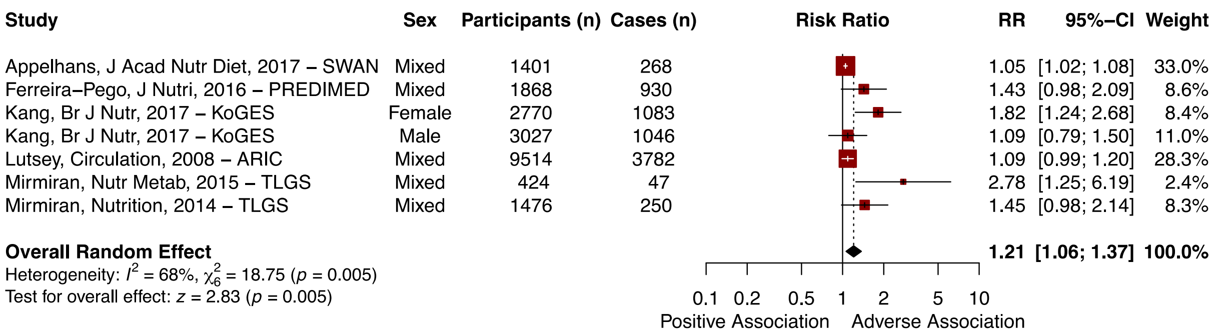

eFigure 1. Relationship between SSB intake and incident MetS.

The black diamond represents the pooled risk estimate. Inter-study heterogeneity was tested using the Cochran Q statistic (Chi^2) at a significance level of $P < 0.10$, and quantified by the I^2 statistic. An I^2 value ≥ 50% is considered to indicate substantial heterogeneity. All results are presented as Relative Risks (RR) with 95% Confidence Intervals where estimable.

© 2020 Semnani-Azad et al. *JAMA Network Open*.

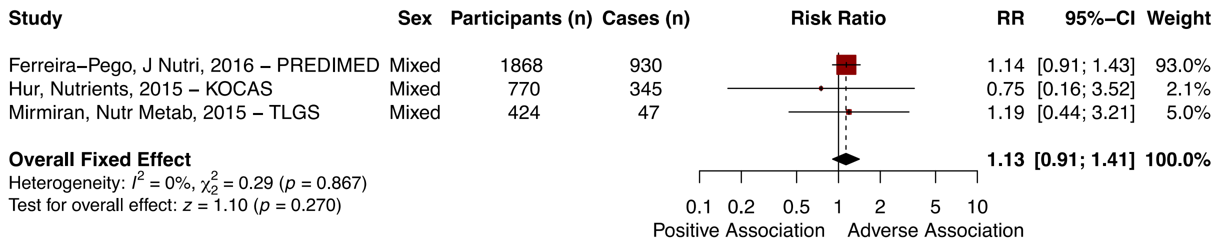

eFigure 2. Relationship between mixed fruit juice intake and incident MetS.

The black diamond represents the pooled risk estimate. Inter-study heterogeneity was tested using the Cochran Q statistic (Chi^2) at a significance level of P < 0.10, and quantified by the I^2 statistic. An I^2 value ≥ 50% is considered to indicate substantial heterogeneity. All results are presented as Relative Risks (RR) with 95% Confidence Intervals where estimable.

© 2020 Semnani-Azad et al. *JAMA Network Open*.

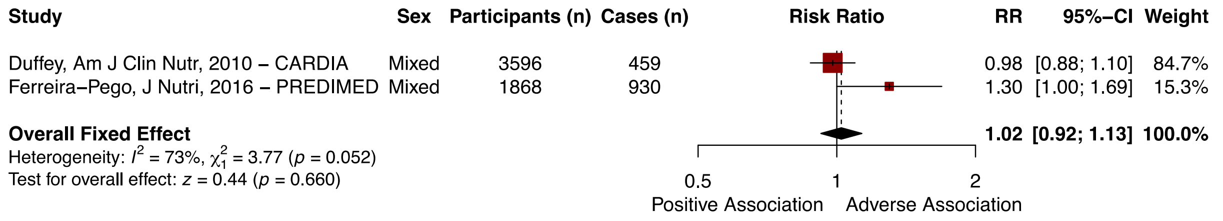

eFigure 3. Relationship between 100% fruit juice intake and incident MetS.
The black diamond represents the pooled risk estimate. Inter-study heterogeneity was tested using the Cochran Q statistic (Chi^2) at a significance level of $P < 0.10$, and quantified by the I^2 statistic. An I^2 value $\geq 50\%$ is considered to indicate substantial heterogeneity. All results are presented as Relative Risks (RR) with 95% Confidence Intervals where estimable.

© 2020 Semnani-Azad et al. *JAMA Network Open*.

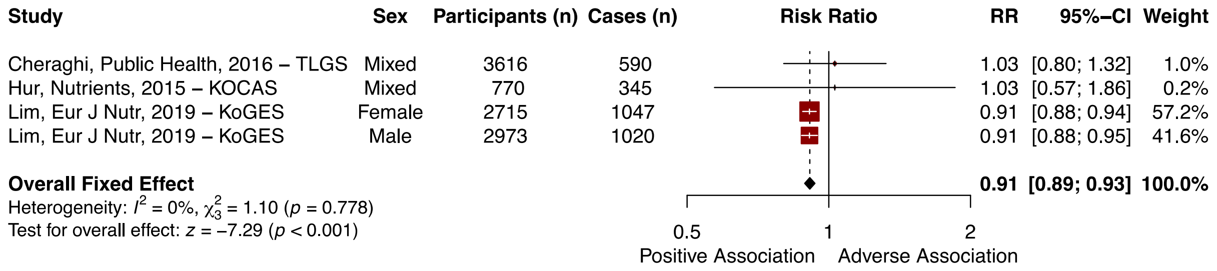

eFigure 4. Relationship between fruit intake and incident MetS.

The black diamond represents the pooled risk estimate. Inter-study heterogeneity was tested using the Cochran Q statistic (Chi^2) at a significance level of $P < 0.10$, and quantified by the I^2 statistic. An I^2 value ≥ 50% is considered to indicate substantial heterogeneity. All results are presented as Relative Risks (RR) with 95% Confidence Intervals where estimable.

© 2020 Semnani-Azad et al. *JAMA Network Open*.

Study	Sex	Participants (n)	Cases (n)	RR	95%-CI	Weight
Babio, J Nutri, 2015 – PREDIMED	Mixed	1868	930	0.77	[0.65; 0.91]	20.2%
Cheraghi, Public Health, 2016 – TLGS	Mixed	3616	590	0.94	[0.84; 1.05]	50.1%
Kim, Br J Nutr, 2017 – KoGES	Female	2651	1063	0.71	[0.58; 0.87]	13.9%
Kim, Br J Nutr, 2017 – KoGES	Male	2859	988	0.67	[0.53; 0.84]	10.8%
Sayon–Orea, BMC Public Health, 2015 – SUN	Mixed	8063	306	0.84	[0.60; 1.18]	5.0%
**Overall Fixed Effect**				**0.83**	**[0.77; 0.90]**	**100.0%**

Heterogeneity: $I^2 = 65\%$, $\chi^2_4 = 11.58$ ($p = 0.021$)
Test for overall effect: $z = -4.75$ ($p < 0.001$)

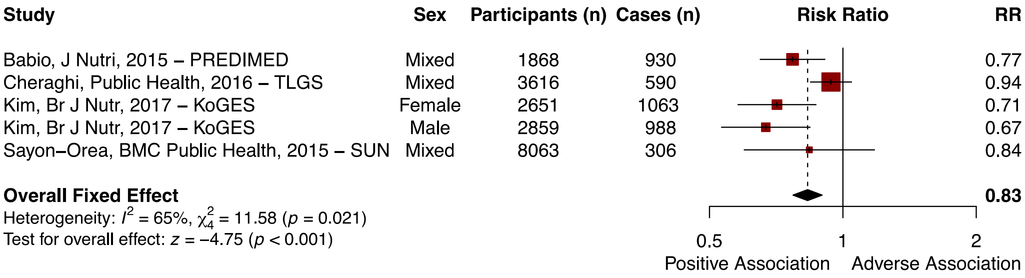

eFigure 5. Relationship between yogurt intake and incident MetS.

The black diamond represents the pooled risk estimate. Inter-study heterogeneity was tested using the Cochran Q statistic (Chi^2) at a significance level of P < 0.10, and quantified by the I^2 statistic. An I^2 value ≥ 50% is considered to indicate substantial heterogeneity. All results are presented as Relative Risks (RR) with 95% Confidence Intervals where estimable.

© 2020 Semnani-Azad et al. *JAMA Network Open*.

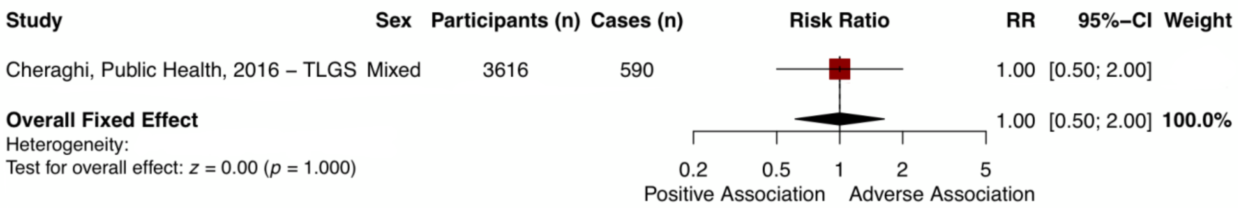

eFigure 6. Relationship between honey intake and incident MetS.

The black diamond represents the pooled risk estimate. Inter-study heterogeneity was tested using the Cochran Q statistic (Chi^2) at a significance level of $P < 0.10$, and quantified by the I^2 statistic. An I^2 value $\geq 50\%$ is considered to indicate substantial heterogeneity. All results are presented as Relative Risks (RR) with 95% Confidence Intervals where estimable.

© 2020 Semnani-Azad et al. *JAMA Network Open*.

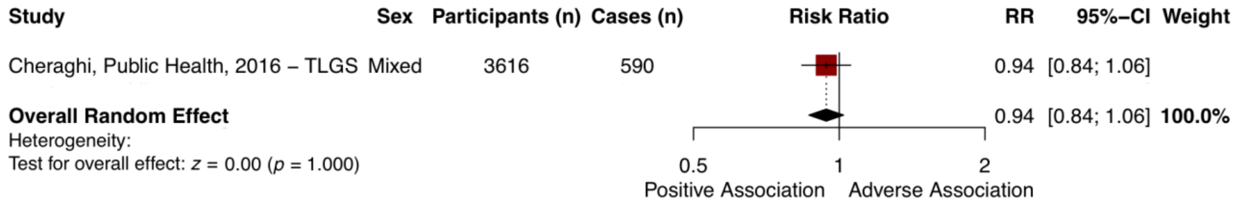

eFigure 7. Relationship between ice-cream intake and incident MetS.
The black diamond represents the pooled risk estimate. Inter-study heterogeneity was tested using the Cochran Q statistic (Chi^2) at a significance level of $P < 0.10$, and quantified by the I^2 statistic. An I^2 value ≥ 50% is considered to indicate substantial heterogeneity. All results are presented as Relative Risks (RR) with 95% Confidence Intervals where estimable.

© 2020 Semnani-Azad et al. *JAMA Network Open*.

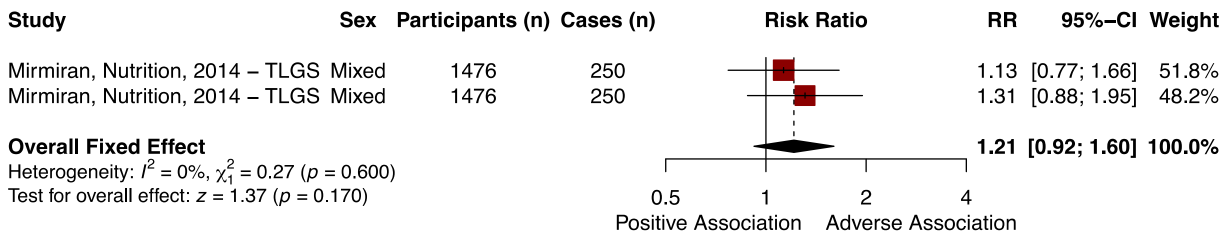

eFigure 8. Relationship between confectionary intake (including cakes, biscuits, chocolate and candies) and incident MetS.

The black diamond represents the pooled risk estimate. Inter-study heterogeneity was tested using the Cochran Q statistic (Chi^2) at a significance level of $P < 0.10$, and quantified by the I^2 statistic. An I^2 value $\geq 50\%$ is considered to indicate substantial heterogeneity. All results are presented as Relative Risks (RR) with 95% Confidence Intervals where estimable.

© 2020 Semnani-Azad et al. *JAMA Network Open*.

eReferences

1. Zhang JY, K. F. What's the relative risk? A method of correcting the odds ratio in cohort studies of common outcomes. *Jama.* 1998;280(19):1690-1691.
2. Bekkering GE, Harris RJ, Thomas S, et al. How much of the data published in observational studies of the association between diet and prostate or bladder cancer is usable for meta-analysis? *Am J Epidemiol.* 2008;167(9):1017-1026.
3. Crippa A, Discacciati A, Bottai M, Spiegelman D, Orsini N. One-stage dose-response meta-analysis for aggregated data. *Stat Methods Med Res.* 2019;28(5):1579-1596.
4. Greenland S, Longnecker MP. Methods for trend estimation from summarized dose-response data, with applications to meta-analysis. *Am J Epidemiol.* 1992;135(11):1301-1309.
5. Orsini N, Li R, Wolk A, Khudyakov P, Spiegelman D. Meta-analysis for linear and nonlinear dose-response relations: examples, an evaluation of approximations, and software. *Am J Epidemiol.* 2012;175(1):66-73.
6. Discacciati A, Crippa A, Orsini N. Goodness of fit tools for dose–response meta-analysis of binary outcomes. *Research synthesis methods.* 2017;8(2):149-160.
7. Assmann G, Guerra R, Fox G, et al. Harmonizing the definition of the metabolic syndrome: comparison of the criteria of the Adult Treatment Panel III and the International Diabetes Federation in United States American and European populations. *Am J Cardiol.* 2007;99(4):541-548.
8. Alberti KG, Eckel RH, Grundy SM, et al. Harmonizing the metabolic syndrome: a joint interim statement of the International Diabetes Federation Task Force on Epidemiology and Prevention; National Heart, Lung, and Blood Institute; American Heart Association; World Heart Federation; International Atherosclerosis Society; and International Association for the Study of Obesity. *Circulation.* 2009;120(16):1640-1645.
9. Appelhans BMB, A. Huang, M. H. Li, H. Janssen, I. Kazlauskaite, R. Avery, E. F. Kravitz, H. M. Beverage Intake and Metabolic Syndrome Risk Over 14 Years: The Study of Women's Health Across the Nation. *Journal of the Academy of Nutrition & Dietetics.* 2017;117(4):554-562.
10. Babio NB-T, N.Martinez-Gonzalez, M. A.Corella, D.Estruch, R.Ros, E.Sayon-Orea, C.Fito, M.Serra-Majem, L.Aros, F.Lamuela-Raventos, R. M.Lapetra, J.Gomez-Gracia, E.Fiol, M.Diaz-Lopez, A.Sorli, J. V.Martinez, J. A.Salas-Salvado, J.Predimed Investigators. Consumption of Yogurt, Low-Fat Milk, and Other Low-Fat Dairy Products Is Associated with Lower Risk of Metabolic Syndrome Incidence in an Elderly Mediterranean Population. *Journal of Nutrition.* 2015;145(10):2308-2316.
11. Cheraghi ZM, P. Mansournia, M. A. Moslehi, N. Khalili, D. Nedjat, S. The association between nutritional exposures and metabolic syndrome in the Tehran Lipid and Glucose Study (TLGS): a cohort study. *Public Health.* 2016;140:163-171.
12. Duffey KJG-L, P. Steffen, L. M. Jacobs Jr, D. R. Popkin, B. M. Drinking caloric beverages increases the risk of adverse cardiometabolic outcomes in the coronary artery risk development in young adults (CARDIA) study. *American Journal of Clinical Nutrition.* 2010;92(4):954-959.
13. Ferreira-Pego CB, N. Bes-Rastrollo, M. Corella, D. Estruch, R. Ros, E. Fito, M. Lluis, S. M. Aros, F. Fiol, M. Santos-Lozano, J. M. Munoz-Bravo, C. Pinto, X. Ruiz-Canela, M. Salas-Salvado, J. Frequent consumption of sugar- and artificially sweetened beverages and natural and bottled fruit juices is associated with an increased risk of metabolic syndrome in a mediterranean population at high cardiovascular disease risk. *Journal of Nutrition.* 2016;146(8):1528-1536.
14. Hur YIP, H.Kang, J. H.Lee, H. A.Song, H. J.Lee, H. J.Kim, O. H. Associations between Sugar Intake from Different Food Sources and Adiposity or Cardio-Metabolic Risk in Childhood and Adolescence: The Korean Child-Adolescent Cohort Study. *Nutrients.* 2015;8(1):31.

© 2020 Semnani-Azad et al. *JAMA Network Open*.

15. Kang YK, J. Soft drink consumption is associated with increased incidence of the metabolic syndrome only in women. *British Journal of Nutrition.* 2017;117(2):315-324.
16. Kim DK, J. Dairy consumption is associated with a lower incidence of the metabolic syndrome in middle-aged and older Korean adults: the Korean Genome and Epidemiology Study (KoGES). *British Journal of Nutrition.* 2017;117(1):148-160.
17. Lim MK, J. Association between fruit and vegetable consumption and risk of metabolic syndrome determined using the Korean Genome and Epidemiology Study (KoGES). *Eur J Nutr.* 2019.
18. Lutsey PLS, L. M.Stevens, J. Dietary intake and the development of the metabolic syndrome: the Atherosclerosis Risk in Communities study. *Circulation.* 2008;117(6):754-761.
19. Mirmiran PB, Z.Delshad, H.Azizi, F. Effects of energy-dense nutrient-poor snacks on the incidence of metabolic syndrome: a prospective approach in Tehran Lipid and Glucose Study. *Nutrition.* 2014;30(5):538-543.
20. Mirmiran PY, E.Asghari, G.Hosseinpour-Niazi, S.Azizi, F. Consumption of sugar sweetened beverage is associated with incidence of metabolic syndrome in Tehranian children and adolescents. *Nutr Metab (Lond).* 2015;12:25.
21. Sayon-Orea CB-R, M.Marti, A.Pimenta, A. M.Martin-Calvo, N.Martinez-Gonzalez, M. A. Association between yogurt consumption and the risk of metabolic syndrome over 6 years in the SUN study. *BMC Public Health.* 2015;15:170.

© 2020 Semnani-Azad et al. *JAMA Network Open*.
